# Adapting an evidence-based intervention for autism spectrum disorder for scaling up in resource-constrained settings: the development of the PASS intervention in South Asia

**DOI:** 10.3402/gha.v8.27278

**Published:** 2015-08-03

**Authors:** Gauri Divan, Syed Usman Hamdani, Vivek Vajartkar, Ayesha Minhas, Carol Taylor, Catherine Aldred, Kathy Leadbitter, Atif Rahman, Jonathan Green, Vikram Patel

**Affiliations:** 1 Sangath, Goa, India; 2 Human Development Research Foundation, Rawalpindi, Pakistan; 3 Institute of Psychiatry, Rawalpindi, Pakistan; 4 Manchester Academic Health Sciences Centre, University of Manchester, Manchester, UK; 5 Institute of Psychology, Health and Society, University of Liverpool, Liverpool, UK; 6 Centre for Global Mental Health, London School of Hygiene and Tropical Medicine, London, UK

**Keywords:** autism spectrum disorder, task-sharing, adaptation, intervention, low-resource setting

## Abstract

**Background:**

Evidence-based interventions for autism spectrum disorders evaluated in high-income countries typically require highly specialised manpower, which is a scarce resource in most low- and middle-income settings. This resource limitation results in most children not having access to evidence-based interventions.

**Objective:**

This paper reports on the systematic adaptation of an evidence-based intervention, the Preschool Autism Communication Therapy (PACT) evaluated in a large trial in the United Kingdom for delivery in a low-resource setting through the process of task-shifting.

**Design:**

The adaptation process used the Medical Research Council framework for the development and adaptation of complex interventions, focusing on qualitative methods and case series and was conducted simultaneously in India and Pakistan.

**Results:**

The original intervention delivered by speech and language therapists in a high-resource setting required adaptation in some aspects of its content and delivery to enhance contextual acceptability and to enable the intervention to be delivered by non-specialists.

**Conclusions:**

The resulting intervention, the Parent-mediated intervention for Autism Spectrum Disorder in South Asia (PASS), shares the core theoretical foundations of the original PACT but is adapted in several respects to enhance its acceptability, feasibility, and scalability in low-resource settings.

South Asia, a region with the largest number of children in the world, is also likely to have the largest number of children with developmental disabilities. Recent epidemiological estimates from India derived from a national study reveals that there are approximately 2 million families with a child with autism spectrum disorder (ASD) between 2 and 9 years (1). The great majority of these, particularly outside major metropolitan centres, have no access to evidence-based care. Previous qualitative work with families and key stakeholders has identified the paucity of skilled personnel to deliver these services and the high costs of specialist-led, centre-based services as an unmet need for families (2, 3). Addressing these barriers requires the delivery of evidence-based treatments, adapted for contextual acceptability (4), using available human resources in family friendly settings.

There is now a significant body of knowledge on the methodology for adaptation of evidence-based treatments, designed and evaluated in high-income countries (HIC), for use in low- and middle-income countries (LMIC) without losing model fidelity or effectiveness. To reduce the treatment gap for mental disorders, major emphasis is on the ‘task-shifting’ to non-specialist health workers, a model pioneered as a pragmatic response to the HIV–AIDS crisis (5). Task-shifting aims at the redistribution of tasks amongst a health force team; specific tasks are moved from highly qualified health workers to those who have fewer qualifications, are trained over a shorter period, but are more available in the community. The authors have extensive experience transferring this approach into adult mental health for diverse conditions, including depression (6), schizophrenia (7, 8), and perinatal depression (9). In this model, specialists play the roles of building capacity, quality assurance, and offering a referral pathway when needed. While a number of studies have demonstrated the feasibility and effectiveness of using non-specialist workers to deliver child and adult mental health interventions (10), a recent systematic review indicates that none of these have been in the context of interventions for developmental disorders in LMIC (11).

Against this background, this paper describes the process of adaptation of an evidence-based intervention for ASD in two South Asian locations. The two key questions that the adaptation process addressed were

1. How should the content of an evidence-based intervention, previously developed and evaluated in an HIC, be adapted to be relevant to the needs of communities in low-resourced contexts?
2. How can the mode of delivery of this intervention be made acceptable and feasible for delivery by a non-specialist worker?

## Methods

### Setting

This adaptation process was undertaken simultaneously in two locations in South Asia. In Goa, a state on the west coast of India, with an equal urban–rural distribution and literacy rates of 80%. The study was implemented by Sangath, a non-governmental research organisation. In Rawalpindi, a north western district in Pakistan with literacy rates of 77%, with an even urban–rural population spread. The study was implemented by the Institute of Psychiatry, Rawalpindi.

### Selection of intervention

The selection of the intervention for adaptation was informed by the method of its delivery and the quality of prior evidence. A parent-mediated delivery method has advantages in this setting notably that parents are a widely available and affordable human resource with important incidental benefits on parental empowerment and parenting practice. The evidence for parent-mediated interventions revealed that social-communication-focused treatments were the only interventions recommended by the UK National Institute for Health and Care Excellence for the early treatment of core symptomatology in ASD (12). These are psychosocial interventions that act to make specific and theoretically based alterations in a child's dyadic communication environment in order to improve the child's social communication, attention, and language. The UK Preschool Autism Communication Therapy (PACT) combines both parent mediation and a social-communication focus (13). The original UK PACT follows a developmental approach to supporting communication, addressing social, pre-linguistic, pragmatic, and linguistic impairments which are present in ASD. It has six stages supporting parents to adapt their communication outputs to their child's abilities. It was delivered during the original trial through fortnightly sessions by speech and language therapists using video-feedback, enabling the parents to identify windows of opportunity to facilitate joint attention and stimulate child intentionality. The fortnightly sessions were continued by 6 monthly sessions to complete a 12-month delivery. Details at www.bbmh.manchester.ac.uk/pact/about/Interventionmanual.pdf

The evidence in support of PACTs effectiveness is derived both from a substantial trial along with recent mediation studies that elucidate the process of its treatment effect (14). The intervention had a rapid and substantial impact after 6 months of treatment on parental communication style with their child with ASD, with a large intervention effect size (ES=1.37) compared to regular UK treatment as usual. This change in parental communicative synchrony then mediates >70% of the improvement observed in the child's communication initiations with the parent (ES 0.5 after 6 months) and 73% of the modest gains in child Autism Diagnostic Observation Schedule (ADOS) symptom score at 13 months (ES 0.24).

### Framework for adaptation

This methodology was based on Phase 1 (formative) and Phase 2 (piloting) of the Medical Research Council framework for the development and adaptation of complex intervention (15). This paper describes the findings from Phase 1 of this framework. The specific steps elaborated here describe the iterative process undertaken by the teams at both sites to develop the Parent-mediated intervention for Autism Spectrum Disorders in South Asia (the PASS intervention). PASS is a communication intervention culturally adapted from the PACT intervention in two South Asian locations for delivery through the task-shifting approach. The theoretical basis of PACT is thus preserved, while a number of distinct modifications to content and the mode of delivery were developed in the light of contextual factors described below.

### Adaptation methods and analysis

Four methods were used; qualitative studies, expert-led simplification of the manual, intervention adaptation workshops, and case series delivered by specialists and non-specialist workers.

#### Qualitative studies

Qualitative research was conducted to assess views on acceptability of the content of the original intervention, the feasibility of its delivery by non-specialists workers, and the strategies to address potential barriers. This included in-depth interviews (IDI, *n*=19) with parents of children with ASD, recruited via key informants in special schools and developmental clinics. Characteristics of the participants are presented in Table 1.

**Table 1:** Characteristics of participants in qualitative studies and case series

Participant characteristics		

	In-depth interviews *n*=20	Case series *n*=17
Primary respondent	Mothers 16; fathers 3; both 1	Mother 16; both 1
Age of child with ASD:	5–9 years: 5 10–15 years: 11 16–18 years: 3	5–9 years: 3 10–14 years: 14
Rural: urban	9: 10	3: 14
Educational level of respondent	Primary school: 3 School completion: 17	Primary school: 1 School completion: 16
Only child	5	5
Family description	Nuclear: 15 Joint^a^: 4	Nuclear: 12 Joint^a^: 5

a Joint family implies an extended family structure where multiple generations or multiple siblings and their families live together.

In addition to these primary data, eight focus group discussions were conducted with two groups of stakeholders working directly or indirectly with children with ASD: pre-school and primary school teachers (*n*=35, rural=18) and special educators (*n*=26, rural=14).

All participants were consented, including for audio recording, by experienced qualitative researchers (a lead supported by a note-taker). The interactions were conducted in the interviewees’ preferred language, in one sitting, lasting 1.5 to 2 hours. A similar process was followed for the focus group discussions. The semi-structured questionnaire included the following explorations: family support for parents, help-seeking behaviours, and opinions of interventions received; the understanding of the development of communication in children; play behaviours of children and intervention-specific enquiries, for example, around the use of a parent-mediated reflective approach; the use of video-feedback; and views around the use of non-specialists. Data analysis was first conducted at the country-level using a framework analysis approach (16, 17) with the topic guide forming the a priori ‘master codes’ framework. Two researchers at each site familiarised themselves with the data and then inductively derived codes from the data within the framework. Researchers across the sites then discussed common and divergent findings and iteratively refined the codes. The results were presented to participants at the Intervention Adaptation Workshops (IAW).

#### Expert-led simplification of the manual

This was undertaken by project specialists which involved simplifying complex terms and ‘key concepts’ for the non-specialist. Similarly, a content review of each stage was conducted to establish clear guidelines on delivery of different strategies in individual sessions. This included creating scripts that explained the theoretical basis of PASS for easier understanding for a non-specialist.

#### Intervention Adaptation Workshops

These were held with local- and national-level experts in the field of ASD. The aim was to elicit expert views on the adapted intervention as well as on the feasibility of task-shifting, the training curriculum, supervision needs, and the characteristics of the non-specialist. A total of 40 experts took part in the intervention development workshops (India, *n*=15; Pakistan, *n*=25), representing diverse backgrounds: speech and language therapists (*n*=11), special educators (*n*=12), developmental paediatricians (*n*=1) occupational therapists (*n*=1), clinical psychologists (*n*=4), psychiatrists (*n*=4), and parents of children with ASD (*n*=7). The workshop proceedings were audio recorded and note-takers transcribed suggestions. The data from the IAW were coded using the themes from the qualitative data analysis along with additional new themes which emerged.

#### Case series

Three regional specialists in child development (a developmental paediatrician, a child psychiatrist, and an occupational therapist) received PACT training in Manchester, UK. This training involved classroom-based sessions, video review, role play, and observing live sessions of PACT sessions over 1 week. On returning to their respective countries, these specialists administered the intervention to eight dyads which were videotaped. Each specialist received Skype supervision by UK-based PACT trainers, both individually and in groups every fortnight. This was followed by the training of non-specialists who then delivered individual practice cases (*n*=9). Hence, the adaptation process included 17 families in the case series prior to the commencement of the trial, characteristics of which are included in Table 1. Inclusion criteria for the case series were children who met criteria for ASD diagnosed by a local professional and on document review. Parents were consented for both delivery of the intervention as well as for video recording.

Information derived from the case series, which warranted changes to the intervention, were discussed in regular supervision meetings; the results were collated into a comprehensive log sheet which included the rationale for the change with adaptations being evaluated in subsequent sessions. Regular discussions between the UK and the South Asian teams resulted in the intervention being adapted incrementally. On completion of a minimum of six sessions in the specialist case series, an independent researcher conducted IDI with the parents at each site, to explore their experience and views on receiving the intervention.

## Results

The data collected through the diverse methods described above were triangulated to address the two key research questions related to the adaptation of PACT. The themes explored for adaptation focused around the content and delivery of the intervention (Table 2).

**Table 2:** Themes explored for adaptation

Themes explored for adaptation		

Master codes	Sub-codes	Themes
Family structures and support	Content	Support for parent to generalise intervention approaches
Help-seeking and opinion on interventions received	Content	Therapy style
Understanding of communication and its development	Content	Expectation from the intervention
Play in families	Content	Acceptability of the use of play in the intervention
Intervention components	Content	Parent agency
		Use of video-feedback
		Goal setting and home work
		Stages of intervention
		Language and metaphor
	Delivery	Training and supervision requirements
		Location of the intervention delivery
Non-specialist characteristics and capabilities	Content and delivery	Stages of intervention for non-specialist delivery
	Delivery	Training requirements for non-specialist

### Adaptation of content

Key areas related to the theoretical framework of the original intervention explored were parents as agents of change, the therapy style, video-feedback, goal setting, and homework which are integral to PACT.

#### Parents as agents of change

The original PACT intervention is parent-mediated, where the therapist works with the parents to enable them to recognise and change their approach of communicating with their child with ASD. We explored views of parent participants in the case series, all of whom had only experienced interventions where therapists worked directly with their child with ASD. An initial reaction of a parent is illustrated below.

To tell you the truth when the whole thing started I had no idea what was going to happen. I was a little sceptical as to whether it was going to be of any use. [Mother, case series]

However, over time one mother described the transfer of skills that was changing her into an expert for her own child.

The positive thing about this therapy is training the mother as a therapist for her child. And there is no better therapist than a mother. [Mother, case series]

All stakeholders interviewed were accepting of this approach. Additionally, some felt that extended or joint family structures, which were part of the local cultural context, could be included in the intervention.

In fact if the session could be done with other members of the family, it would be good [for] all of us to behave the same way with the child. [Special educator, FGD]

PACT in the UK was delivered to only one parent. During the case series, attempts were made to deliver the intervention to more than one parent or family member. However, opposing individual learning styles made this increasingly difficult for a non-specialist to handle independently. As a result, PASS was adapted to be delivered to only one parent, who was then encouraged to share their home programme with others in the family.

#### Therapy style

PACT strategies are imparted to parents through a reflective process, with the therapist adapting instructions based on the learning style of the individual parent. A concern by the experts was that most parents in the local context are used to a directive approach from clinicians compared to reflective thinking. However, diverse parents in the case series were able to adjust to the reflective mode and were able to understand the impact their changed behaviours were making.

And that's what (was) told to me that you have to change yourself, you forget what he is doing, try to understand him, instead of expecting him to understand you. And that's what clicked for me to tell you the truth. [Mother, case series]

The collaborative nature of the therapeutic alliance was initially a potential challenge. However, as sessions progressed parents became actively involved in the reflective nature of the dialogue during the video-feedback.

#### Use of video-feedback

PACT is based on video-feedback, which is an effective teaching methodology for adult learning. Though stakeholders understood the benefits of using the video-feedback, the potential stress of being on camera was raised by a number of them. An initially anxious parent illustrated its importance:

It helps me because sometimes you are so involved in what you're saying and what you are doing that you don't notice what the child is doing or feeling because he really can't tell you …. Only his body movements, gestures, the way he turns his head is actually enough to tell you; but we are so used to (instructing). So when I saw it I actually realized the importance of being in sync with what he wants or doesn't want. [Mother, case series]

Another parent described how the video-feedback gave clarity to the strategies parents were being asked to adopt.

Seeing myself in video made a difference. When you observe yourself you realize what you are doing and how pushy you are towards your child. [Mother, case series]

#### Goal setting and homework

In each fortnightly PACT session, parent and therapist agree on mutually agreed goals which the parent is then expected to practice every day for 30 min. During the case series, a detailed home programme was prepared at the end of each session and these were collated, forming an individualised parent manual. The experts at the IAW suggested labelling the daily sessions as *home-practice* versus the original *homework* to distinguish this from their child's school ‘home-work’. They also shared the difficulty many families have in putting new skills into practice. It was suggested that the non-specialist worker should help parents in identifying opportunities to consolidate and generalise skills learnt during the sessions. The home practice proved challenging for some of the parents, while others found it easier compared to other interventions they had experienced.

Sometimes I am lazy, means the half an hour practice session is the most difficult to tell you the truth. [Mother, case series]

However, as this parent began to see the impact of her changes during the play session she was then able to fit the ‘practice’ into her son's routines by joining him in play versus creating a ‘time to play’. Maintaining a daily diary of home practice was impractical for most parents, and this was seen as a potential challenge in a region of varying literacy.

#### Stages of intervention

The initial stages were deemed by experts in the IAW to be simple enough for non-specialist delivery, but concerns were raised for the last stage of PACT which is dependent on the knowledge of language development.

#### Use of play

PACT uses a naturalistic approach which is set up around ‘playing with the child’. A box of toys is taken to each session to help the child and parent share a focus. The main concern was cultural, and addressed the lack of importance given to adults playing with a child and the potential unease with this kind of approach.

They (children) mostly play with their friends. Usually in our village, parents don't play (with their children). [Mother, IDI]

Most interventions for ASD in the local context focused on teaching and learning skills; a play-based intervention seemed novel and unusual. However, the advantages of having an intervention which was less didactic and fun were also recognised by some participants in the IAW. A parent in the case series remarked on the pivotal role of play during the sessions:

I was given training in the child development centre to give him new words. But in this (PASS) I was told to do things that make the child happy … when he is happy (and playing), that is the time when the child wants to (communicate). When you get him interested, when you fulfil his interest (by playing the way he wants); then I saw improvement. [Mother, case series]

An exploration of common games played and toys used was part of the qualitative work. Rural parents in both countries did describe casual play with accessible materials such as sticks, leaves, water play, and household items.

#### Language and metaphor

Technical language used in the original PACT manual was simplified to support the non-specialist understanding of key concepts and to ease understanding and explanations for parents. For example, ‘sharing enjoyment’ was further clarified in the manual to explain its components in the following manner.

Sharing enjoyment can sometimes be easy to notice or may need closer observation. It is reflected in three ways during the play session; by the facial expressions the parent shows (e.g., smiles versus a worried expression); by the parents actions (e.g. if the parent copies what their child is doing, or quietly observes their child instead of redirecting him with their gestures or actions) or by the words a parent may use (e.g., praise or positive comments instead of instructing their child). [Expert led simplification]

Experts suggested analogies should be culturally and locally appropriate. For example, in the PACT manual the concept of mapping words is described by an analogy of thinking of ‘post-its’ on every object; however, a parent provided an analogy of each object having its own ‘microphone’, which shouts out its name. Similarly, the concept of ‘demands’ being made on the child was simplified to ‘pressure’ which one specialist adapted into a culturally specific analogy shown below:

Suppose you were cooking a meal and your mother-in-law is watching you and giving you continuous instructions. How would you feel? Compare it to her letting you cook without bothering you. When would you be more relaxed? [Specialist, case series]

### Mode of delivery

#### Non-specialist agents

PACT was delivered by speech and language therapists, a resource that is scarce in low-resource settings. An integral part of the adaptation process was to develop an intervention which was deliverable by a non-specialist, trained and supervised by a specialist. This process of task-shifting was discussed with all stakeholders who were open to this idea, as long as the person was from the local community, well trained in understanding autism, competent, and empathetic of the challenges of families. Though experts recommended higher educational qualifications, parents who received the intervention during the case series had no difficulty with the education level of the worker delivering PASS.

I don't have much (opinion) about education but if he has knowledge in this then it is enough. It is not that all those who are educated only are intelligent. Some are intelligent on the basis of their talent also. [Mother, case series]

Only one parent (in Pakistan) had a specific recommendation of the gender of the non-specialists, preferring women, who would have easier access to mothers in their home.

#### Training and supervision to attain competency in intervention delivery

The original PACT training in the UK has a standard 2-day training which is supported by one-on-one post course supervision sessions led by speech and language therapists. In the context of a non-specialist delivering this complex intervention, most experts felt that trainees should receive a minimum of 4-week training which would include information on child development, ASD and other disabilities, and the practical training on building therapeutic alliances and observation skills. It was suggested that the training should be led and supervised by a specialist with a clear understanding of ASD as well as the intervention. All stakeholders also expressed the need to establish clear referral guidelines for aspects of child health and co-existing ASD-specific problems beyond the expertise of the non-specialists. During the training and delivery of case series by the non-specialists, an objective methodology for rating competencies of the non-specialist was also developed.

#### Delivery setting

The original PACT intervention was delivered predominantly in a clinical setting with an option for delivery at home. In the study locations, the stigma of having a child with special needs was mentioned as a deterrent to home delivery.

… because some don't want to show that their child has autism. Some people are such whose neighbours also don't know that the child is with autism. Some don't tell. So such problems will come, no one will accept in the beginning. [Special educator, FGD]

In India, the special educational facility was suggested as a setting which could avoid the stigma of an intervention delivered at home. Facility-based interventions were also preferred in Pakistan, where it was customary for the parents to visit a therapist.

## Discussion

The need to deliver high-quality evidence-based ASD interventions with relatively low resources is critical in LMIC and has also been recognised as a priority in HIC (18). To the best of our knowledge, this paper describes the first effort to systematically adapt an evidence-based intervention for ASD to ensure its feasibility and acceptability of its delivery by non-specialist health workers in two South Asian settings. Our main findings are that the PACT intervention is acceptable and relevant to the needs of the local communities and its relatively low intensity makes it is easily transferable. The method of delivery by non-specialist health workers was also found to be feasible and acceptable.

In essence, the adapted intervention is based on the same theoretical construct as PACT and utilises a naturalistic approach to scaffolding and developing communication skills in the child with ASD. However, the resulting PASS required adaptation in some aspects of its content and delivery to enhance contextual acceptability and to enable the intervention to be delivered by non-specialists.

In the cultural context of grandparents or nannies being the primary caregivers, flexibility in the adult targeted for the intervention was introduced. Despite concerns of experts about the ‘reflective’ nature of the intervention, parents were able to adapt to this approach with ease during video-feedback. Parents were also able to engage readily in the ‘play sessions’, using standardised materials containing locally familiar household objects and toys. Homework was renamed as ‘home-practice’ to make it seem less arduous and keeping it distinct from the child's after-school responsibilities. The adapted intervention allocates a defined time during each session when a parent is encouraged to identify opportunities where PASS strategies can be practiced. The treatment goals are pragmatic; parents are guided to understand that the aim of PASS is to improve communication and not the language acquisition of their child. The last stage of PACT, involving an in-depth understanding of language development, has been re-designed to be co-delivered by the specialist supervisors. Contextually, appropriate metaphors and analogies have been included to illustrate the concept of communication and the details of specific strategies.

The characteristics of the non-specialist has been identified as a graduate without any specific child development training but possessing a range of non-academic ‘soft’ skills, including good communication skills and willingness to travel to the homes of families. The 10-day foundation training package is tailored to include concepts of typical child development; an introduction to disabilities; counselling approaches; opportunities for observing typically developing children and children with ASD; exercises to support the scaffolding of trainee's observation skills; and the details of the PASS stages. Progression to practice cases is dependent on trainees passing a knowledge test. The first two sessions of the practice cases are co-delivered with a supervisor, with the non-specialist leading the second session. Four weeks after the commencement of training, when each trainee had led at least one practice session under supervision, they undergo an objective competency test. This linked PASS Competency Measure includes a knowledge and skills component and is an essential pre-requisite for independent delivery by the non-specialist. Subsequently, provisions are made for ‘top-up’ training as dyads transition to later stages of the intervention. The flexibility in the original PACT intervention with respect to the setting of the delivery has also been retained, with Pakistan preferring a facility-based intervention. An important component of the initial engagement with the family has been designed to be conducted by the specialist supervisor with the aim of clarifying the goals of PASS as well as explaining the supervisory framework. All seven non-specialists across the sites had no difficulty in establishing therapeutic alliances with families. The adaptation contains clear guidelines explicitly detailing potential concerns (e.g. behavioural problems) that need to be referred to supervisors, to safeguard non-specialists from being consulted on issues beyond the scope of their training.

The principal limitation of this study is its restricted cultural testing. The adaptation process was conducted in only two areas of a very disparate subcontinent. However, one strength is the use of mixed methods which included eliciting practice-based evidence from experts drawn from throughout the region. The adaptation process described in this paper has resulted in the PASS intervention which retains the key theoretical constructs of the original PACT intervention whilst being adapted for delivery by non-specialists in two different low-resource settings in South Asia.

The pathways to evidence-based care for families affected by ASD in LMIC involve a difficult journey for most parents (2, 3), often leaving parents either with no care or confused by the choices in the absence of a clear evidence base. This paper describes the systematic adaptation of an evidence-based intervention for ASD developed in a high-resource setting for delivery through the process of task-shifting to non-specialist health workers in South Asia, reallocating the role of the scarce resource of specialists to primarily be that of trainers and supervisors.

## Ethical standards

The work done in this project received appropriate ethical clearance from the institutional review boards of all the participating organisations in accordance with the ethical standards laid down in the 1964 Declaration of Helsinki and its later amendments. All adult participants gave their informed consent prior to their inclusion in the study.
